# Supplementary figures and images for: New insight into the divergent responses of plants to warming in the context of root endophytic bacterial and fungal communities
Source: PeerJ. 2021 May 26;9:e11340. doi: 10.7717/peerj.11340 (PMC8164412; doi:10.7717/peerj.11340)

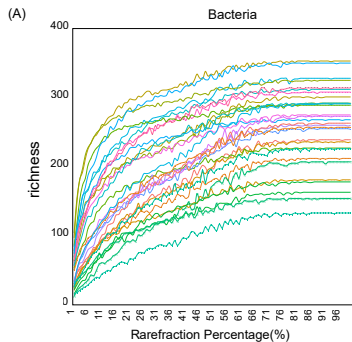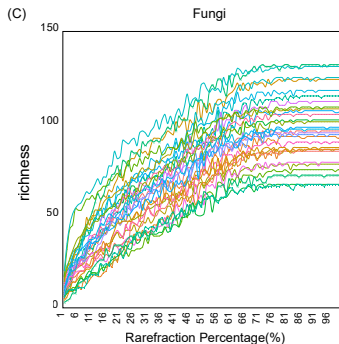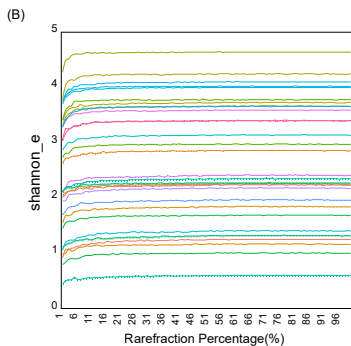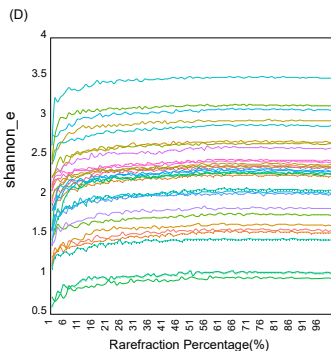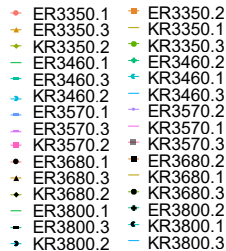

Supplement: Figure S1 — ER indicated Elymus nutans root, KR indicated Kobresia pygmaea root. 3,350, 3,460, 3,70, 3,680, 3,800 indicated elevations at 3,350 m, 3,460 m, 3,570 m, 3,680 m and 3,800 m, respectively. 1, 2, 3 represent repetitions. [file peerj-09-11340-s001.pdf]

(A)

## Bacteria

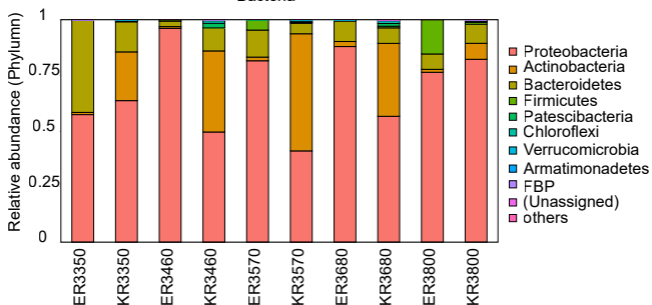

(B)

## Fungi

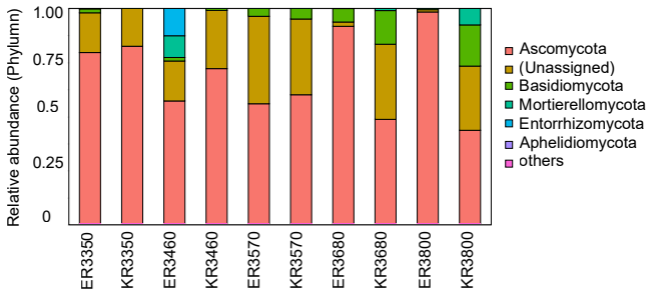

Supplement: Figure S2 — ER represent E. nutans root; KR represent K. pygmaea root; the number represent elevations. For example, ER3350 indicate E. nutans root at the elevation of 3,350 m; KR3350 indicate K. pygmaea root at the elevation of 3,350 m. The same as below. [file peerj-09-11340-s002.pdf]

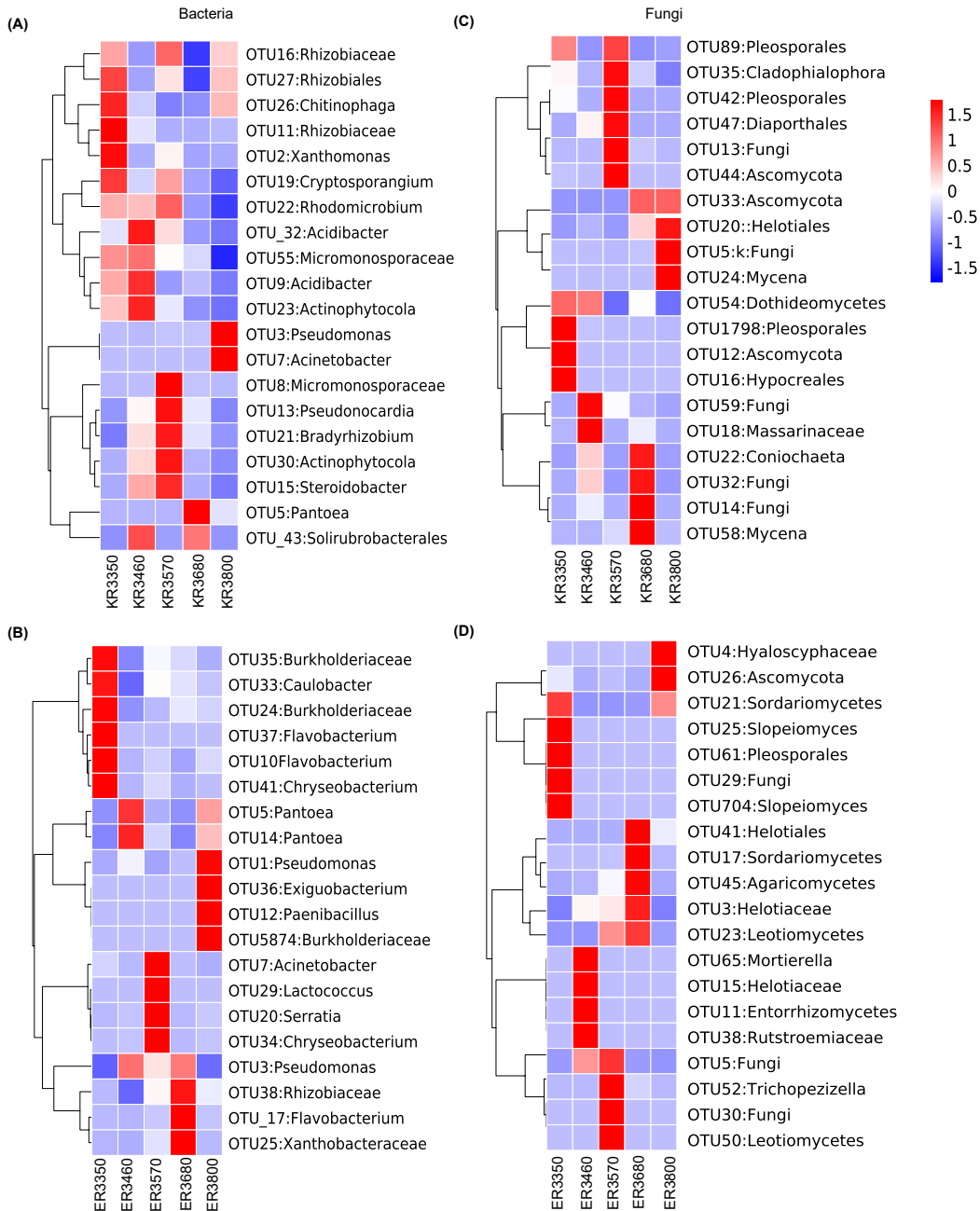

Supplement: Figure S3 — ER represent E. nutans root; KR represent K. pygmaea root; the number represent elevations. For example, ER3350 indicate E. nutans root at the elevation of 3,350 m; KR3350 indicate K. pygmaea root at the elevation of 3,350 m. The same as below. [file peerj-09-11340-s003.pdf]

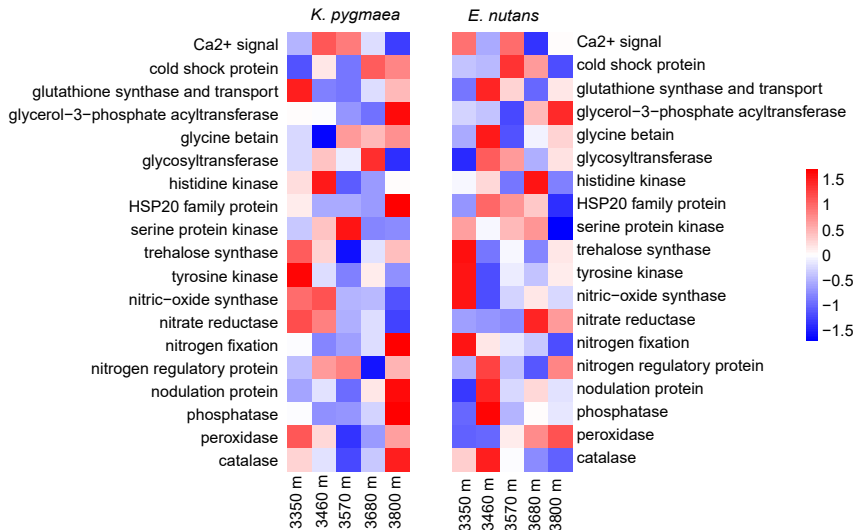

Supplement: Figure S4 [file peerj-09-11340-s004.pdf]
